# Use of the intrascope channel stent release technique using a novel pigtail-type plastic stent

**DOI:** 10.1055/a-2445-8148

**Published:** 2024-11-08

**Authors:** Masafumi Watanabe, Kosuke Okuwaki, Tomohisa Iwai, Kai Adachi, Akihiro Tamaki, Taro Hanaoka, Chika Kusano

**Affiliations:** 1 38088 Gastroenterology, Kitasato University School of Medicine, Sagamihara, Japan

Endoscopic retrograde cholangiopancreatography (ERCP) with plastic stent placement is well established for managing biliary obstruction. Although pigtail-type plastic stents have a lower migration risk compared with straight plastic stents, their design necessitates that there is a longer length protruding from the papilla. To maintain an adequate distance between the papilla and the endoscope, adjustments in the operating angle or forceps elevator manipulation are critical during the deployment of pigtail plastic stents, making this a technically challenging procedure.

The Piglet Stent (Olympus Medical Systems, Tokyo, Japan) represents a novel design that integrates a pushing catheter and pigtail-type plastic stent with a unique claw-shaped structure. This innovative design allows the plastic stent to remain attached to the pushing catheter even after the inner sheath and guidewire have been removed ( Fig. 1 ). Consequently, if the inner sheath is inadvertently released within the intrascope channel, the stent can be readily placed by simply pushing it out. This makes the intrascope channel stent release technique 1 easy, with minimal movement for angle adjustments and forceps elevator use. In this case, we introduce an intrascope channel stent release technique that leverages this feature of the novel pigtail-type plastic stent.

**Fig. 1:**
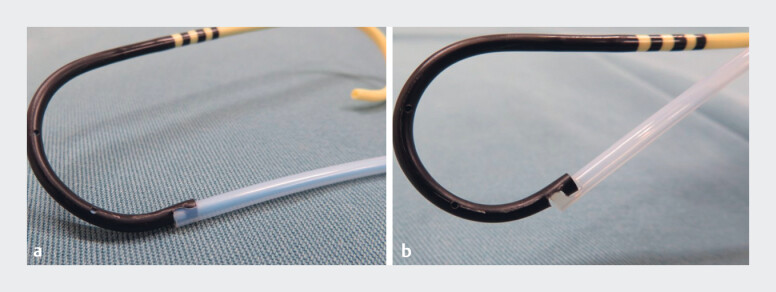
Photographs of the novel pigtail-type plastic stent showing: **a** the plastic stent with the inner sheath in place (the pushing catheter and the plastic stent are integrated by the claw-shaped structure); **b** the plastic stent without the inner sheath – even after removal of the inner sheath and guidewire, the claw-shaped structure maintains the connection between the pushing catheter and the plastic stent.

The patient was a 79-year-old woman with a history of gastric cancer surgery (Billroth I reconstruction). Stent replacement was performed for plastic stent obstruction secondary to hilar cholangiocarcinoma. Owing to challenges in maintaining endoscope stability during plastic stent deployment, the intrascope channel stent release technique was attempted. The Piglet Stent was successfully advanced to the target area and released within the intrascope channel. The pushing catheter was carefully advanced without altering the scope position, ensuring that the tip remained stationary, thereby facilitating the successful sequential placement of three plastic stents ( Video 1 ).

The intrascope channel stent release technique is performed with the novel pigtail plastic stent, allowing successful stent deployment with minimal adjustment of angles and forceps elevator manipulation.Video 1

In conclusion, the intrascope channel stent release technique using the novel pigtail plastic stent offers a viable alternative that overcomes the traditional challenges associated with the design of standard pigtail plastic stents.

Endoscopy_UCTN_Code_TTT_1AR_2AZ

## Notes

Endoscopy E-Videos https://eref.thieme.de/e-videos
*E-Videos* is an open access online section of the journal *Endoscopy* , reporting on interesting cases and new techniques in gastroenterological endoscopy. All papers include a high-quality video and are published with a Creative Commons CC-BY license. Endoscopy E-Videos qualify for HINARI discounts and waivers and eligibility is automatically checked during the submission process. We grant 100% waivers to articles whose corresponding authors are based in Group A countries and 50% waivers to those who are based in Group B countries as classified by Research4Life (see: https://www.research4life.org/access/eligibility/ ). This section has its own submission website at https://mc.manuscriptcentral.com/e-videos .
